# The value of Tablets as reading aids for individuals with central visual field loss: an evaluation of eccentric reading with static and scrolling text

**DOI:** 10.1111/opo.12296

**Published:** 2016-04-07

**Authors:** Robin Walker, Lauren Bryan, Hannah Harvey, Afsane Riazi, Stephen J. Anderson

**Affiliations:** ^1^ Department of PsychologyRoyal HollowayUniversity of LondonEghamUK; ^2^ NeurosciencesSchool of Life and Health SciencesAston UniversityBirminghamUK

**Keywords:** eccentric viewing, low vision, macular degeneration, reading, scrolling text

## Abstract

**Purpose:**

Technological devices such as smartphones and tablets are widely available and increasingly used as visual aids. This study evaluated the use of a novel app for tablets (MD_evReader) developed as a reading aid for individuals with a central field loss resulting from macular degeneration. The MD_evReader app scrolls text as single lines (similar to a news ticker) and is intended to enhance reading performance using the eccentric viewing technique by both reducing the demands on the eye movement system and minimising the deleterious effects of perceptual crowding. Reading performance with scrolling text was compared with reading static sentences, also presented on a tablet computer.

**Methods:**

Twenty‐six people with low vision (diagnosis of macular degeneration) read static or dynamic text (scrolled from right to left), presented as a single line at high contrast on a tablet device. Reading error rates and comprehension were recorded for both text formats, and the participant's subjective experience of reading with the app was assessed using a simple questionnaire.

**Results:**

The average reading speed for static and dynamic text was not significantly different and equal to or greater than 85 words per minute. The comprehension scores for both text formats were also similar, equal to approximately 95% correct. However, reading error rates were significantly (*p* = 0.02) less for dynamic text than for static text. The participants’ questionnaire ratings of their reading experience with the MD_evReader were highly positive and indicated a preference for reading with this app compared with their usual method.

**Conclusions:**

Our data show that reading performance with scrolling text is at least equal to that achieved with static text and in some respects (reading error rate) is better than static text. Bespoke apps informed by an understanding of the underlying sensorimotor processes involved in a cognitive task such as reading have excellent potential as aids for people with visual impairments.

## Introduction

Smartphones, tablets and electronic readers often incorporate basic features such as image enlargement and high‐contrast screens that can be used as effective low vision aids.^1^ The capabilities of these devices can be further extended by the development of bespoke apps that are tailored towards specific visual impairments.^2^ For example, tablets and smartphones can present text in a range of formats including dynamically as horizontally scrolling lines (similar to a news ‘ticker’) or as a serial stream of words at a single location (rapid‐serial visual‐presentation – *RSVP*). The use of dynamic text presentation methods could aid reading in individuals with a central field loss (CFL)^3^, ^4^, ^5^, ^6^ as exhibited in macular degeneration (MD).

Individuals with macular degeneration often make use of their relatively preserved peripheral vision and self‐select a preferred area of their eccentric retina (preferred retinal loci, or PRL).^7^, ^8^, ^9^ The use of the eccentric viewing technique can develop spontaneously within 6 months of disease onset,^10^ and has been associated with improvements in reading speed.^11^ Reading with dynamic formats such as RSVP and scrolling lines involves a different pattern of eye movements^12^, ^13^ to the stereotypical pattern used for reading normally,^14^ and could enhance eccentric reading in people with a central field loss.^4^, ^6^, ^15^

The effective use of eccentric viewing for reading requires the reader to be able to hold an eccentric gaze position (at their PRL) and this ability can be compromised in people with MD,^16^ as can their oculomotor control.^9^, ^17^ A technique called the ‘steady‐eye’ strategy, where the reader holding a steady eccentric viewing position while moving the page of text from right‐to‐left in front of their eyes,^18^ may reduce the demands on the oculomotor system. The eccentric viewing and steady‐eye techniques can be combined and there is some evidence that these strategies reduce reading difficulties.^19^, ^20^ The use of dynamic text formats, such as horizontally scrolling sentences^6^ and RSVP,^15^ can potentially mitigate the difficulties encountered in eccentric reading by reducing the demands to make eye movements. Faster reading rates have been observed with horizontal drifting text compared with static text^6^ and RSVP^4^ in cases of CFL. A study employing a simulated central scotoma to mimic CFL reported a reduction in reading errors and improved adherence to eccentric viewing with scrolling text compared with static text.^5^

An advantage of electronic devices is that they can enable text to be presented with dynamic formats and this may have wide‐ranging benefits for people with low vision. Furthermore, other potential textual characteristics (e.g. font size, word spacing, line spacing, colour) can also be manipulated. The present study examined reading performance and subjective reading experience with static and scrolling text in individuals with macular disease. Participants read single sentences of either static or scrolling text presented on a tablet (iPad2) and were instructed to adopt an eccentric viewing strategy.

## Methods

### Participants

The participants were all recruited from the membership of the Macular Society (UK), which actively promotes the eccentric viewing technique. As such it was expected that participants would, in general, be aware of this technique. As they were not recruited from a clinical setting they were not ‘patients’ as such and we did not have access to their clinical details (e.g. whether they were receiving treatment or not). The inclusion criteria were: a diagnosis of binocular macular degeneration (wet or dry); over 18 years of age, binocular distance acuity between 0.30 and 0.80 (Mean 0.55, S.D. 0.24) logMAR (Snellen equivalents 6/12 or 20/40 to 6/40 or 20/135) and English as primary language. The exclusion criteria were: inability to read 24‐point font; ocular co‐morbidity; dyslexia and any cognitive impairment. Informed consent was collected from all participants prior to the study, as approved by Royal Holloway Psychology departmental and NHS ethical review (reference, 14LO0047).

### Materials

The sentences used in the assessment of reading performance consisted of 40 sentences from the MNRead compilation,^6^ which are all of a standard length of 46 characters (excluding spaces) or approximately 10–12 words long (e.g. ‘Every Tuesday the jazz band took requests to play songs’*)*. A double space was included between each word to reduce visual crowding.^21^ Twenty sentences were presented dynamically (horizontally‐scrolling) on a tablet device (iPad2) in Arial 24 point black font on a yellow background using the MD_evReader app.^2^ On the basis of an earlier pilot study,^2^ the scrolling speed was set at a comfortable rate of approximately 180 characters/minute for all participants. Twenty single sentences were presented in static format (same font size, colour as for scrolling) using the SlideShark^R^ (Brainshark Inc.; https://www.slideshark.com/) presentation app that allows text to be manipulated and presented on an iPad (similar to Microsoft Powerpoint^R^; www.microsoft.com/en-gb). At the end of each sentence the participant was asked a simple comprehension question (e.g. ‘Was it a rock band that took requests?’). A digital mp3 recorder recorded the participants reading aloud for later off‐line scoring. Binocular reading acuity was measured using the MNRead Acuity Chart.^6^

### User experience questionnaire

Following the assessment of reading performance, participants completed a short user evaluation questionnaire (Appendix 1). Items probed their explicit awareness and active use of the eccentric viewing and steady eye techniques as well as their subjective experience of reading with the MD_evReader app, along with factors that would deter them from using such devices.

### Procedure

**Figure 1:**
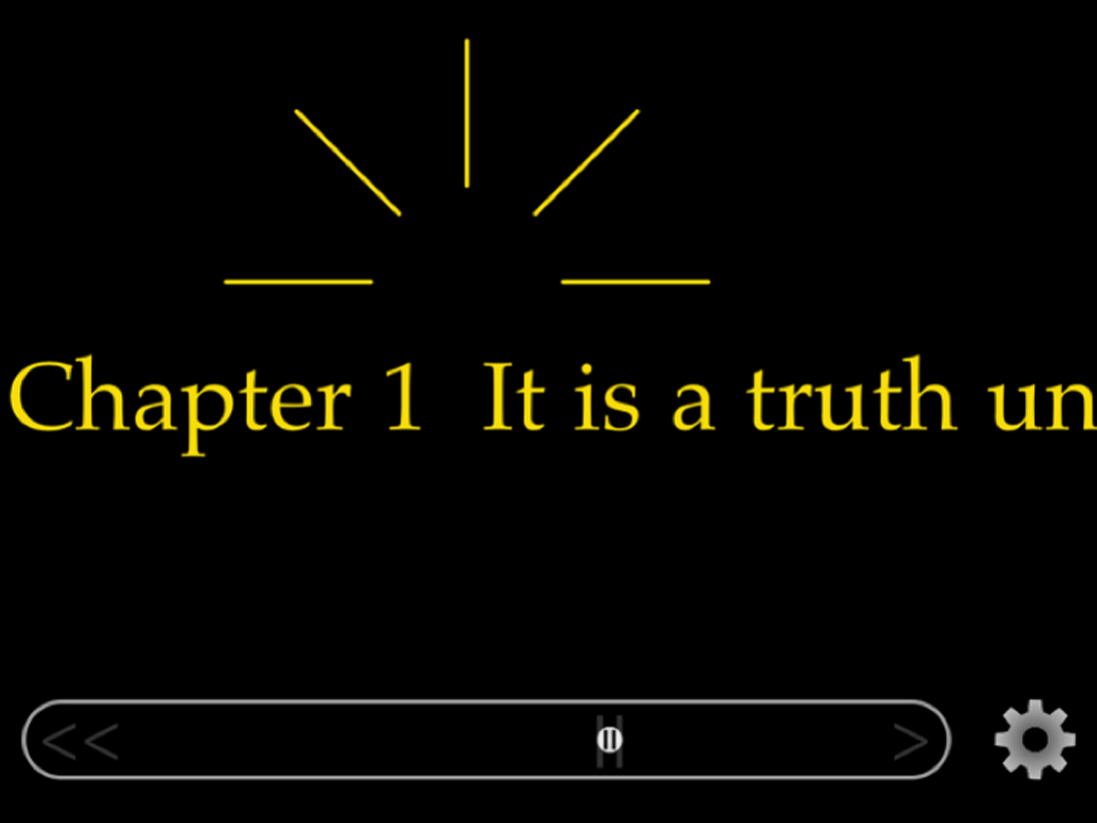
Main screen of MD_evReader showing fixation stimulus positioned above a single line of (scrolling) text. The trackpad (bottom of screen) is used to control the speed of scrolling.

Binocular visual acuity was assessed using the MNRead Acuity chart at a distance of 40 cm (or 30 cm if required). The eccentric viewing and steady‐eye strategies were explained to participants and they were asked to adhere to these strategies as much as possible when reading. The preferred retinal locus for each participant was assessed using an Amsler chart: ten participants had received training in eccentric viewing (provided by the Macular Society) and were aware of their ideal PRL. For scrolling text a movable fixation marker (controlled by the MD_evReader app (*Figure* 1) was then positioned on the tablet screen as a landmark for gaze position to be held such that the text was located at the participant's PRL (e.g. if the PRL was in the lower right visual field the eccentric fixation stimulus was positioned above and to the left of the text). For static text, participants were instructed to read whilst holding an eccentric viewing position at their PRL.

Participants first performed a short practice session in which they read three scrolling and three static sentences presented on an iPad2 tablet held upright using a stand at a viewing distance of approximately 40 cm. If required, each participant could make adjustments to the position of the movable fixation stimulus following this practice. The eccentric fixation stimulus was presented in the scrolling text condition only; participants were instructed to read static text whilst moving their gaze along the line at their preferred eccentric viewing position. For the assessment, participants were asked to read 10 scrolling sentences and then 10 static sentences. This sequence was repeated with a further two sets of 10 sentences to counterbalance the text presentation methods. A short break was given between each block of sentences. Participants were encouraged to read whilst holding an eccentric viewing position and to adopt the steady‐eye strategy with scrolling text. Recording of the participants reading started when they first vocalised, not when the sentence appeared on the screen. At the end of the testing session participants completed the user evaluation questionnaire (with assistance from the researcher for reading the questions if required). Reading performance measures for scrolling and static text formats were examined using non‐parametric statistics (Wilcoxon signed‐ranks test), and the median was used to describe the Likert‐scale questionnaire scores.

The audio recording enabled reading speed and errors to be quantified after the session. A sentence was deemed to contain errors if the participant omitted words, added additional words, or read a word incorrectly (even if they subsequently corrected the error). After each sentence, participants were asked a comprehension question and were required to respond with either “Yes” or “No”. An incorrect answer was scored as zero, while a correct answer was scored as one. Reading speed was recorded for each sentence with a stopwatch. It should be noted that for scrolling text participants typically waited for some seconds for words to appear on the screen before they started to read aloud.

## Results

Twenty‐six (23 female) adults aged from 42 to 93 years (mean age = 75.8 years) volunteered to take part in the study. Of these, 14 had attended a higher education institute, including 11 at University/Polytechnic level. All reported a prior diagnosis of macular disease as follows: left eye diagnoses included: 14 wet AMD, 10 dry AMD and two Stargardt disease; right eye diagnoses included: 10 wet AMD 14 dry AMD and two juvenile forms (Stargardt's disease). Average length of diagnosis was 110 months (range 8–516 months). Ten participants (38.5%) had prior knowledge of the eccentric viewing technique and stated that they used the strategy for tasks, including reading (average time using EV was 35.5 months).

Following the initial assessment of the PRL, twenty‐two participants positioned the fixation stimulus (a cross) above the line of text, while four positioned the stimulus below the sentence. The first trial of static and scrolling text were excluded, with each participant's reading assessed on the remaining 19 sentences for each condition (*Table* 1).

**Table 1:** Average comprehension scores (%), and the % of sentences read without errors for static and scrolling lines of text

	Static text (%)	Scrolling text (%)
Comprehension score	94.9	94.3
Percentage of sentences read without errors	72.7	77.9

Reading performance for static and scrolling single sentences. Average comprehension (percentage comprehension questions correctly answered) and the average percentage of sentences read without errors.

### Comprehension and Error rates

Comprehension performance was high for both the scrolling and static text formats (94.3% and 94.9% respectively), and a comparison between conditions was not significant (Wilcoxon‐signed‐ranks test *Z* = −0.48, *p* = 0.63, *r* = 0.03). The overall number of reading errors was low (*Median* scrolling = 0.21, static = 0.24) and the difference between the two conditions was not significant (Wilcoxon‐signed‐ranks test *Z* = −1.35, *p* = 0.18, *r* = 0.84). The proportion of sentences read without errors was, however, significantly greater for scrolling text (77.9%) than static text (72.7%), Wilcoxon‐signed‐ranks test *Z*=−2.26, *p* = 0.02, *r* = 0.82).

### Reading Speed

The average time taken to read was 7.2 s for static sentences (S.D. 4.5, range 2.8–17.0 s) and 7.8 s for scrolling sentences (S.D. 4.1, range 3.2s–18.0 s), which equates to reading speeds of approximately 91.6 and 84.6 words per minute respectively. A t‐test confirmed that reading speed was comparable in the two text formats [*t*(25) = 1.94, *p* = 0.064].

### User experience questionnaire

Following the reading assessment participants completed a short questionnaire designed to probe their prior knowledge and stated use of the eccentric viewing technique and also their subjective experience of reading scrolling text presented using the MD_evReader app. The questionnaire items and the median responses on a five‐point Likert scale are reported in *Appendix* 1.

## Discussion

Reading performance in 26 adults with binocular macular degeneration and a central field defect was assessed using a tablet with an app developed to enhance eccentric reading.^2^ Participants were instructed to read using the eccentric viewing technique, and performance was assessed for single sentences of either static or horizontally‐scrolling text. Reading performance was good overall with a high average reading speed observed in both conditions (overall mean = 84–92 w.p.m), along with excellent comprehension rates (95% correct). Average reading error rates and comprehension scores were comparable across presentation formats, and a small reduction in the number of sentences read without errors was observed for dynamic scrolling text. Our data shows that reading performance with scrolling text is at least equal to that achieved with static text and in some respects (reduced error rates) is better than with static text. Given reading scrolling text is an unusual situation and many of the participants reported not being familiar with the eccentric viewing technique, the high comprehension and low reading error rates observed for dynamic text demonstrates the potential benefits of bespoke apps for tablet devices as low vision aids for individuals with a CFL.

The questionnaire ratings of user experience of reading scrolling text with the MD_evReader app were positive (*Appendix* 1). The majority of participants rated the MD_evReader highly as a reading aid and three‐quarters said that it would encourage them to read more than they do at present. A similar percentage reported that their reading experience with the MD_evReader app was equally good or better than their current method. Although it is plausible that the positive ratings may be subject to a degree of acquiescence bias, the overall positive responses to questions focused on reading scrolling text presented on tablet are encouraging. Reading horizontally‐scrolling text is an unusual situation and combining this with eccentric viewing and steady‐eye strategies in a single assessment session is not ideal for evaluating its potential as a low‐vision aid. Despite these limitations reading speed and comprehension were comparable across formats and reading errors were reduced with scrolling text. Reading performance with dynamic text formats presented on eReaders and Tablets may benefit from interventions, such as perceptual learning, which has been shown to produce tangible improvements after a small number of practice sessions.^22^

The limitations of the present study include the lack of prior experience of eccentric viewing for some participants and the potential unreliability in the assessment of the participants’ PRL with an Amsler grid. Future studies could investigate the effects of perceptual learning,^23^ combining eccentric viewing with static and a wider range of dynamic formats (scrolling and RSVP) over a longer period of time, with added performance measures (e.g. minimum font size, duration of comfortable reading). User feedback could be collected using features incorporated into the app rather than relying on a separate questionnaire.

## Disclosure

The authors report no conflicts of interest.

## Appendix 1 User experience questionnaire

Participants completed a questionnaire (Table A1) devised to evaluate user experience of reading using the MD_evReader iPad app. The questionnaire was completed following the assessment of reading performance. The researcher read out each question and explained the nature of the response required (either on a 5‐point Likert scale, or with a yes/no response) and recorded the responses.

**Table A1:** Results from the user evaluation questionnaire showing the questions and the median ratings (0–4 Likert scale) and interquartile range (IQR), and yes/no responses to questions 10 and 11

	User evaluation questionnaire	Median ratings (IQR 25th ‐ 75th percentiles)
1	How much do you use the eccentric fixation technique when reading? 0 – None of the time, 4 – All of the time	1 (0 ‐ 2.75)
2	How much do you use the steady eye technique when reading? 0 – None of the time, 4 – All of the time	0 (0 ‐ 3)
3	How useful did you find the eccentric fixation point to aid reading the text? 0 – Not useful, 4 – Very useful	2.5 (1 ‐ 3)
4	Did you feel that the app helped you to read more easily than usual? 0 – Not at all, 4 – A lot	3 (2 ‐ 3)
5	How did you find reading with the app compared to reading the static text sentences? 0 – More difficult, 4 – Much easier	2.5 (2 ‐ 3)
6	In terms of ease of use, how did using the app compare to your usual method for reading? 0 – More difficult, 4 – Much easier	3 (2 ‐ 3.75)
7	In terms of your reading experience, how did using the app compare to your usual method for reading? 0 – Much worse, 4 – Much better	2.5 (2 – 3)
8	How likely would you be to use this app as a long‐term aid for reading? 0 – Very unlikely, 4 – Very likely	4 (2 – 4)
9	How would you rate the app as a reading aid overall? 0 – Very poor, 4 – Very good	4 (3 – 4)
10	Would an app like this encourage you to read more? Yes or No	Yes = 21 No = 5
11	If you feel you would be unlikely to use the app as a long‐term aid for reading, what reason would you say most describes why this is?	
	Cost (Yes/No) Not easy to use (Yes/No) Prefer existing visual aids (Yes/No)	0/26 1/25 4/22

The questionnaire items are shown in Table A1. Items 1 and 2 aimed at examining the participants’ prior knowledge of and stated use of the eccentric viewing and steady‐eye strategies for reading. Item 3 related to the movable fixation stimulus presented on the iPad screen that acted as a guide for holding an eccentric gaze location with dynamic scrolling text. Items 4–9 related to the participants’ subjective preferences of reading with scrolling text when using the MD_evReader app. Question 10 asked if participants thought that an app like the MD_evReader would encourage them to read more than at present and the last three items examined issues that may deter them from using an app, including cost, ease of use and possession of other reading aids.

### Findings and summary

Less than half (10/26) of the participants included in the study were explicitly aware of eccentric viewing (either informed by Macular Society eccentric viewing trainers, or literature). It is quite likely, however, that participants may have spontaneously developed the use of an eccentric preferred retinal location (Crossland 2005)^10^ despite their lack of acknowledged awareness of the strategy. For those participants who stated they had experience of the eccentric viewing strategy, the average length of time since they first adopted the use of the technique was 13.6 months (range 2–132 months). The median questionnaire ratings for questions 1 and 2 relating to use of eccentric viewing and steady‐eye techniques for reading were low (1 and 0 respectively) and show a large degree of variability, which illustrates the variability in prior knowledge of eccentric viewing.

Items 4–7 examined the participant's experience of reading scrolling text presented on the iPad using the MD_evReader app. Median scores indicated users found it easier to read dynamic text presented with the app than when reading normally, and when compared to reading static sentences and with their usual preferred reading method. Scores for ease of use and experience of reading with the app were also high compared with participants’ usual reading method. The participants’ evaluations of the iPad app for reading were positive: a median of 4.0 was given for the question that asked how likely they would be to use the app as a long‐term reading aid. When asked how they would rate the app overall, the median score for participants was 4.0, with 90% of participants rating the app positively (rating of 3 or 4). Participant age was negatively correlated with their average approval rating of the app, with older age being associated with a lower rating (ρ= ‐48, *p* = 0.02). Of all participants, 84% agreed that the app would encourage them to read more. One reason given why it was unlikely they would use the app was that they preferred their existing reading aid (4/26 cases).

Although the positive evaluations of reading experience when using the iPad app are encouraging, they should be interpreted with a degree of caution as they may be subject to the influence of acquiescence bias. Participants verbally reported their ratings directly to the research assistant and it is quite likely that they would be more likely to make their responses more positive than was actually the case. Bespoke apps like the MD_evReader have the potential to enhance further the basic features of tablet devices and enable methods of text presentation, including dynamic and RSVP which are not available on standard eReaders. The cost of developing and purchasing apps is relatively inexpensive and they offer excellent potential as innovative low vision aids.

## References

[1] Crossland MD , Silva RS & Macedo AF . Smartphone, tablet computer and e‐reader use by people with vision impairment. Ophthalmic Physiol Opt 2014; 34: 552–557. 25070703 10.1111/opo.12136

[2] Walker R . An iPad app as a low‐visual aid for people with macular disease. Brit J Ophthalmol 2013; 97: 110–112. 23087416 10.1136/bjophthalmol-2012-302415

[3] Bowers AR , Woods RL & Peli E . Preferred retinal locus and reading rate with four dynamic text presentation formats. Optom Vis Sci 2004; 81: 205–213. 15017180 10.1097/00006324-200403000-00013

[4] Fine EM & Peli E . Scrolled and rapid serial visual presentation texts are read at similar rates by the visually impaired. J Opt Soc Am A Opt Image Sci Vis 1995; 12: 2286–2292. 7500210 10.1364/josaa.12.002286

[5] Harvey H & Walker R . Reading with peripheral vision: a comparison of reading dynamic scrolling and static text with a simulated central scotoma. Vis Res 2014; 98: 56–60. 10.1016/j.visres.2014.03.009 24680772

[6] Legge GE , Ross JA , Luebker A & LaMay JM . Psychophysics of reading 8. The Minnesota low‐vision reading test. Optom Vis Sci 1989; 66: 843–853. 2626251 10.1097/00006324-198912000-00008

[7] Fletcher DC & Schuchard RA . Preferred retinal loci relationship to macular scotomas in a low‐vision population. Ophthalmol 1997; 104: 632–638. 10.1016/s0161-6420(97)30260-7 9111255

[8] Von Noorden GK & Mackensen G . Phenomenology of eccentric fixation. Am J Ophthalmol 1962; 53: 642–661. 13926696 10.1016/0002-9394(62)91987-6

[9] Whittaker SG , Budd J & Cummings RW . Eccentric fixation with macular scotoma. Investigative Ophthalmol Vis Sci. 1988; 29: 268–278. 3338884

[10] Crossland MD , Culham LE , Kabanarou SA *et al* Preferred retinal locus development in patients with macular disease. Ophthalmology 2005; 112: 1579–1585. 16087239 10.1016/j.ophtha.2005.03.027

[11] Nilsson UL , Frennesson C & Nilsson SEG . Patients with AMD and a large absolute central scotoma can be trained successfully to use eccentric viewing, as demonstrated in a scanning laser ophthalmoscope. Vis Res 2003; 43: 1777–1787. 12818347 10.1016/s0042-6989(03)00219-0

[12] Buettner M , Krischer C & Meissen R . Characterization of gliding text as a reading stimulus. B Psychonomic Soc 1985; 23: 479–482.

[13] Potter MC . Rapid serial visual presentation (RSVP): a method for studying language processing. New methods in reading comprehension research In: New Methods in Reading Comprehension Research (Kieras D. & Just M editors), Erlbaum: Hillsdale, 1984; 118: pp. 91–118.

[14] Rayner K . Eye movements in reading and information processing: 20 years of research. Psychol Bull 1998; 124: 372–422. 9849112 10.1037/0033-2909.124.3.372

[15] Rubin GS & Turano K . Low‐vision reading with sequential word presentation. Vis Res 1994; 34: 1723–1733. 7941378 10.1016/0042-6989(94)90129-5

[16] Crossland MD , Culham LE & Rubin GS . Fixation stability and reading speed in patients with newly developed macular disease. Ophthalmic Physiol Opt 2004; 24: 327–333. 15228511 10.1111/j.1475-1313.2004.00213.x

[17] Timberlake GT , Mainster MA , Peli E , Augliere RA , Essock EA & Arend LE . Reading with a macular scotoma I. Retinal location of scotoma and fixation area. Invest Ophth Vis Sci 1986; 27 : 1137–1147. 3721792

[18] Watson G & Berg R . *Near training techniques* In: Understanding Low Vision, (Jose R, editor), American Foundation for the Blind: New York, 1983; pp. 317–362.

[19] Nilsson UL & Nilsson SEG . Rehabilitation of the visually handicapped with advanced macular degeneration. Doc Ophthalmol 1986; 62: 345–367. 2942377 10.1007/BF00168266

[20] Palmer S , Logan D , Nabili S & Dutton GN . Effective rehabilitation of reading by training in the technique of eccentric viewing: evaluation of a 4‐year programme of service delivery. Brit J Ophthalmol 2010; 94: 494–497. 19822918 10.1136/bjo.2008.152231

[21] Blackmore‐Wright S , Georgeson MA & Anderson SJ . Enhanced text spacing improves reading performance in individuals with macular disease. PLoS One 2013; 8: e80325. 24244676 10.1371/journal.pone.0080325 PMC3823704

[22] Chung ST . Improving reading speed for people with central vision loss through perceptual learning. Investigative Ophthalmol Vis Sci 2011; 52: 1164–1170. 10.1167/iovs.10-6034 PMC3053100 21087972

